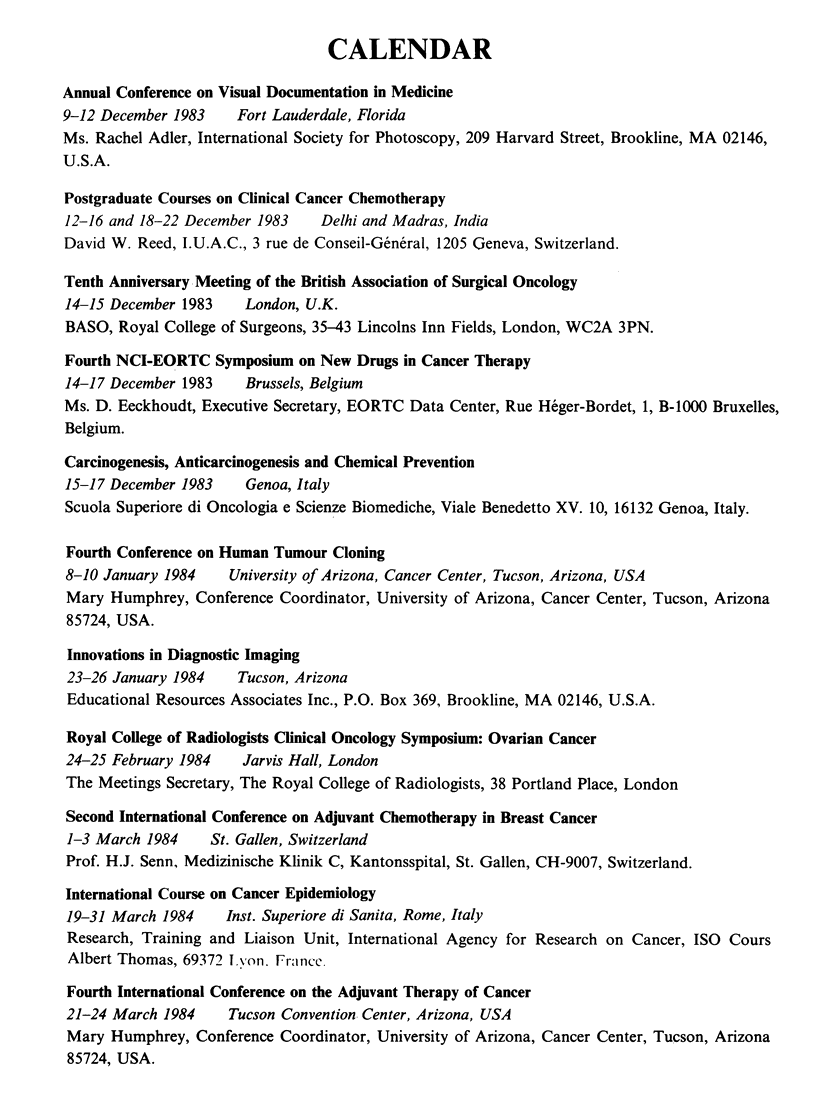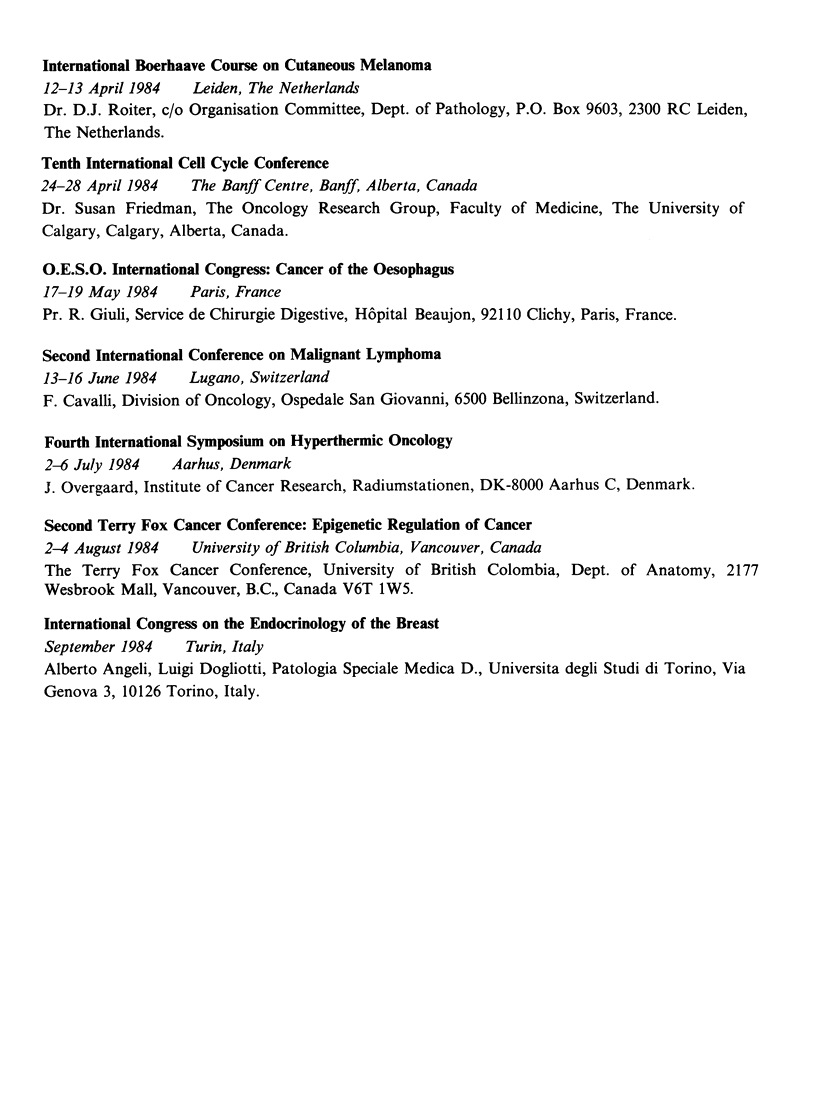# Calendar

**Published:** 1983-12

**Authors:** 


### CALENDAR

**Annual Conference on Visual Documentation in Medicine**
*9–12 December 1983*    *Fort Lauderdale, Florida*
Ms. Rachel Adler, International Society for Photoscopy, 209 Harvard Street, Brookline, MA 02146, U.S.A.

**Postgraduate Courses on Clinical Cancer Chemotherapy**
*12–16 and 18–22 December 1983*    *Delhi and Madras, India*
David W. Reed, I.U.A.C., 3 rue de Conseil-Général, 1205 Geneva, Switzerland.

**Tenth Anniversary Meeting of the British Association of Surgical Oncology**
*14–15 December* 1983    *London, U.K.*
BASO, Royal College of Surgeons, 35–43 Lincolns Inn Fields, London, WC2A 3PN.

**Fourth NCI-EORTC Symposium on New Drugs in Cancer Therapy**
*14–17 December* 1983    *Brussels, Belgium*
Ms. D. Eeckhoudt, Executive Secretary, EORTC Data Center, Rue Héger-Bordet, 1, B-1000 Bruxelles, Belgium.

**Carcinogenesis, Anticarcinogenesis and Chemical Prevention**
*15–17 December 1983*    *Genoa, Italy*
Scuola Superiore di Oncologia e Scienze Biomediche, Viale Benedetto XV. 10, 16132 Genoa, Italy.

**Fourth Conference on Human Tumour Cloning**
*8–10 January 1984*    *University of Arizona, Cancer Center, Tucson, Arizona, USA*
Mary Humphrey, Conference Coordinator, University of Arizona, Cancer Center, Tucson, Arizona 85724, USA.

**Innovations in Diagnostic Imaging**
*23–26 January 1984*    *Tucson, Arizona*
Educational Resources Associates Inc., P.O. Box 369, Brookline, MA 02146, U.S.A.

**Royal College of Radiologists Clinical Oncology Symposium: Ovarian Cancer**
*24–25 February 1984*    *Jarvis Hall, London*
The Meetings Secretary, The Royal College of Radiologists, 38 Portland Place, London

**Second International Conference on Adjuvant Chemotherapy in Breast Cancer**
*1–3 March 1984*    *St. Gallen, Switzerland*
Prof. H.J. Senn, Medizinische Klinik C, Kantonsspital, St. Gallen, CH-9007, Switzerland.

**International Course on Cancer Epidemiology**
*19–31 March 1984*    *Inst. Superiore di Sanita, Rome, Italy*
Research, Training and Liaison Unit, International Agency for Research on Cancer, ISO Cours Albert Thomas, 69372 Lyon, France.

**Fourth International Conference on the Adjuvant Therapy of Cancer**
*21–24 March 1984*    *Tucson Convention Center, Arizona, USA*
Mary Humphrey, Conference Coordinator, University of Arizona, Cancer Center, Tucson, Arizona 85724, USA.

**International Boerhaave Course on Cutaneous Melanoma**
*12–13 April 1984 Leiden, The Netherlands*
Dr. D.J. Roiter, c/o Organisation Committee, Dept. of Pathology, P.O. Box 9603, 2300 RC Leiden, The Netherlands.

**Tenth International Cell Cycle Conference**
*24–28 April 1984 The Banff Centre, Banff, Alberta, Canada*
Dr. Susan Friedman, The Oncology Research Group, Faculty of Medicine, The University of Calgary, Calgary, Alberta, Canada.

**O.E.S.O. International Congress: Cancer of the Oesophagus**
*17–19 May 1984 Paris, France*
Pr. R. Giuli, Service de Chirurgie Digestive, Hôpital Beaujon, 92110 Clichy, Paris, France.

**Second International Conference on Malignant Lymphoma**
*13–16 June 1984 Lugano, Switzerland*
F. Cavalli, Division of Oncology, Ospedale San Giovanni, 6500 Bellinzona, Switzerland.

**Fourth International Symposium on Hyperthermic Oncology**
*2–6 July 1984 Aarhus, Denmark*
J. Overgaard, Institute of Cancer Research, Radiumstationen, DK-8000 Aarhus C, Denmark.

**Second Terry Fox Cancer Conference: Epigenetic Regulation of Cancer**
*2–4 August 1984 University of British Columbia, Vancouver, Canada*
The Terry Fox Cancer Conference, University of British Colombia, Dept. of Anatomy, 2177 Wesbrook Mall, Vancouver, B.C., Canada V6T 1W5.

**International Congress on the Endocrinology of the Breast**
*September 1984 Turin, Italy*
Alberto Angeli, Luigi Dogliotti, Patologia Speciale Medica D., Universita degli Studi di Torino, Via Genova 3, 10126 Torino, Italy.